# Beta Thalassemia Major in a Developing Country: Epidemiological, Clinical and Evolutionary Aspects

**DOI:** 10.4084/MJHID.2013.002

**Published:** 2013-01-02

**Authors:** Mohamed Bejaoui, Naouel Guirat

**Affiliations:** Centre national de greffe de moelle osseuse, Tunis Tunisia

## Abstract

Beta-thalassemia major (TM) remains to be one of the major health problems particularly in developing countries. Tunisia is a part of the Mediterranean countries mostly affected by this disease which is highly concentrated in small towns in families with low-income earners. The main objectives of this study are to provide a description of the demographic, clinical features and transfusion-related complications in patients with TM living in Tunisia. A standardized questionnaire was sent to clinicians throughout 33 different medical institutions caring for thalassemic patients. 391 transfusion dependant thalassemic patients with a median age of 10.7 years (range 3 months- 31 years) were included in the study. The majority originated from the north west of the country. A moderate iron overload between 1501 and 2500 ng/ml was found in 61patients, while 81 patients (26.9%) had a ferritin level more than 2500 ng/ml and greater than 5000ng/ml in 21 patients (6.9%). 51 patients died from complications related to their disease. Heart failure was the main cause of death. The incidence of cardiac, endocrine, and infectious complications will be reviewed. Preventive measures such as health education, carrier screening and premarital screening remain the best ways for lowering the incidence of these diseases, which might be reflected in financial saving, social s and health benefits.

## Introduction

TM is one of the most common hereditary diseases in Tunisia. Although its true incidence is unknown, it is estimated that 4.48 % of Tunisian population harbour thalassemic trait.^1^ It remains a health problem in our country either for the clinicians who follow TM patients or to the patients themselves.

Lifelong red blood cells transfusion remains the main treatment for severe homozygous beta thalassemia even if hematopoietic stem cell transplantation is more and more utilized being the only definitive curative therapy for homozygous thalassemia.^2^ In fact there are numerous risks and considerable morbidity associated with chronic transfusion therapy.^3^ Each unit of blood carries a small but definite risk of transmitting infections.^4^ In addition, repeated blood exposure can induce alloimmunization to erythrocytes antigens, leading to difficulties in identifying compatible blood. Finally, long term erythrocyte transfusions inevitably lead to severe iron overload with its related complications involving the liver, the heart and the endocrine organs.^5^ This study was aimed at assessing the present epidemiological profile and the clinical features of TM major patients living in Tunisia.

## Materials and Methods

The study was performed as a retrospective and descriptive observation. A standardized questionnaire was sent to clinicians throughout 33 different medical institutions in Tunisia caring for thalassemic patients. The questionnaire was used to collect demographic and clinical data (family history, age, sex, origin, consanguinity, age at diagnosis, age at the first blood transfusion and outcome); markers of iron overload (ferritin level, and/or serum iron); transfusion therapy and transfusions complications related to haemochromatosis (cardiac siderosis evaluated according to the results of the electrocardiogram and cardiac Doppler ultrasound, endocrinological complications), chelating therapy (date of onset, type of chelation, modalities).

Statistical analysis: Fisher’s exact test was used to assess intergroup significance between categorical variables, and Student’s *t*-test was used to determine differences between continuous variables. The statistical analysis was carried out using software (SPSS version 11.5). A p value <0.05 was considered statistically significant.

## Results

Three hundred and ninety one transfusion dependant thalassemic patients [174(44.5%) females and 217 males (55.4%); mean age 10.7 years; range 3 months to 31 years] were included in the study. Origin was determined in 382 cases. The majority of the patients come from the west of the country; central west 117cases (30%) and North West 107cases (27.3%). It is important to note that the large migration flows from the western towns of Tunisia to the capital that contributed to the higher appearance of TM in Tunis. However, among the studied patients 22% were from small towns and cared in Tunis, only 5% of them live in the capital. Consanguinity was found in 244 among 324 studied patients (75.3%).

Most of the patients 325/391(83.1%) were transfused at intervals of 3–4 weeks; 51 patients (13%) were transfused at an interval of 5–8 weeks and 15 patients (3.8%)poorly controlled and were transfused only in an emergency situation.). 126 patients (32.2%) received filtrated blood cells while only 14 patients (3.5%) received non phenotypically red blood cells. Transfusion-transmitted infections with hepatitis B and C viruses were diagnosed respectively in 2.3 % and 6.1% of patients. No infection with human immunodeficiency virus was found. A serum antibody screening was realized systematically before each transfusion for 209patients (53.4%) and unevenly for 107 patients (27.3%). Alloantibodies were detected in 26 patients (8.2%) and belonged mainly to rhesus system (76.9%). Direct antiglobulin test was performed in 300 patients. Of the total 300 patients 105 (35%) developed auto antibodies.

Chelation therapy was administered to 341 patients (87.2%). Only the third of the patients received chelation therapy before they had completed their 3^rd^ year and 33.3% of patients had started chelation at the age of 3 or later. Deferoxamine was the most commonly used iron chelator (224/391=57.3%) administered initially by intramuscular injection in 191 cases (85.2%) and 99 patients (51.8%) continued to use this route. Subcutaneous bolus injections were used for 61 patients and with infusion pumps for 64 other patients. At the time of the first survey deferasirox was administered to 16.7% and deferiprone was administered only to 12.4% of patients. Serum ferritin levels were evaluated in 301 patients. The majority of the patients revealed high ferritin levels.

Fifty- nine patients (19.6%) had serum ferritin levels between 1001 – 1500 ng/ml. A moderate iron overload between 1501 and 2500ng/ml was found in 61patients, while 81 patients (26.9%) had serum ferritin values above 2500 ng/ml and levels higher than 5000 ng/ml were determined in 21 patients (6.9%). The evaluation of serum ferritin levels revealed considerable differences depending on chelating therapy (Table 1).

During two years of the study, a gradual decrease in mean ferritin levels was observed in patients treated with deferasirox versus deferoxamine either administered by pump or independently by injections (p = 0.001 and 0,02 respectively). Moreover, patients treated with Deferasirox presented with lower mean ferritin levels than those receiving deferiprone (p< 0.001). Analyses of the three treatment groups deferoxamine, deferasirox and deferiprone administered alone showed strong differences between serum ferritin concentrations and treatment groups (p=0.006). Cardiac disorders occurred in 63 among 318 studied patients (19.8%). Mean age of onset was 16.9 years (3–30). The youngest patient was died from heart failure related to severe anemia.. Heart failure defined as dyspnea and/or peripheral edema with sinus tachycardia on electrocardiography without signs or symptoms of current or recent infection, thyroid disorders, autoimmune diseases or exposure to cardiotoxic agents was found in 34 among 63 studied patients (53.9%)

Pulmonary hypertension was found in 12.5% of the patients while arrhythmias were found only in five among the 63 studied patients. Mean serum ferritin level was significantly higher in patients with cardiopathy compared to those without cardiopathy (3000 versus 2053 ng/ml; p=0.005).

Delayed puberty was the most common endocrine complication in this study. It was observed in 46/90 (51.1%) of the patients. Among the 90 patients, 48 were males and 42 were females. Delayed puberty is more common in boys [26/48 boys (54.1%)] than in girls [20/42 (47.6%)]. Growth retardation was seen in 64/239 (26.7%) of patients with mean age 12.9 years (3–22years). Thyroid function studies were available for 262 patients. Evidence of reduced organ activity was present in 18 cases (6.8%). Thirteen of 309 patients (4.2%) had diabetes mellitus, all diagnosed after the age of 9 years. The mean age at the time of diagnosis was 15.1 years. Mean serum ferritin level was found to be a contributing factor to endocrine disorders (Table 2).

Splenectomy was performed in 201/372 (54.0 %) of the patients [181 (90%) total splenectomy and 20 (9.9%) subtotal splenectomy]. Mean age of patients at splenectomy was 7 years. Routine vaccination against Streptococcus pneumonia, H. influenzae type b, and Neisseria meningitides had been received by 98.3%, 72 % and 85.2% of patients, respectively. Prophylactic antibiotics with benzylpenicillinbenzatine had been prescribed for 53.5 % of the patients however only 35.8% were treated with oral penicillin.

Cholelithiasis was observed in 7.9% of the patients (n = 31). Mean age at detection by ultrasonography was 13 years (range 3–22). Twenty four (77.4%) of the 31 patients with cholelithiasis had undergone cholecystectomy (6.1% of the whole population). Mortality occurred in 51/391 (13%) studied patients. The mean age of death was 10.48 years (2–21). Heart failure was the major leading cause of death: 20/51 (39%), followed by severe infections: 13/51(25.4%). Five patients (9.8%) died of severe anemia. A significant difference was observed in the rate of mortality related to cardiopathy, type of chelation therapy and serum ferritin levels (Table 3). 33 deaths were reported during the deferoxamine treatment while only four deaths were reported during oral chelating therapy (p-value=0.001). All patients were followed up at UHC. Mean patient age at onset of the oral chelating treatment was 17 years (12–18years). Two patients were treated with deferasirox while two others had received deferiprone. Mean total period of oral chelating therapy period was two years. No correlation was observed between mortality and splenectomy.

## Discussion

Tunisia is a part of the Mediterranean countries mostly affected by thalassemia, one of the most common genetic diseases in the world. The prevalence of TM is especially high in countries where there are close family marriages.^6^ Geographic distribution in this study demonstrated that TM is highly concentrated in small towns particularly in the western part of the country where there are marriages between close relatives.

Blood transfusion and iron chelation remain the cornerstone of treatment for patients with TM^7^. However, there are several risks associated with chronic blood transfusions; firstly the risk of transmitting infections. In addition, long term erythrocyte transfusions inevitably lead to severe iron overload. Finally, repeated blood exposure can induce immunization, leading to difficulty in identifying compatible blood. Hepatitis C and B viruses are the most common infection agents transmitted via transfusions and routine screening is performed for these agents throughout the world. In contrast to other studies,^8^,^9^ the rate of transfusion transmitted infections with HCV is lower in our population. Alloantibodies were detected in 8.2% of our patients. These findings were in accordance with the results of Ahrens et al.^5^ but were lower than those reported by Singer et al.^2^ who reported a rate of alloimmunization of 44% among transfusion dependant thalassemia patients of predominantly Asian origin. This difference may be explained by several factors, family donation is frequently solicited in Tunisia explaining the antigenic homogeneity between donor’s antigens system and recipient’s RBC antigens. Another factor that could contribute to the low frequency found in the present study might be the early stage of the first blood transfusion for the majority of our patients. It has been thought that transfusion at an early age may offer some protection against red cell alloimmunization because of immune tolerance for young children.^3^,^10^ In the present study the most frequently detected alloantibodies were anti rhesus system. This finding is in accordance with other reported data.^11^,^12^ The prevalence of autoimmunization in multitransfused thalassemic patients in our population is high as compared to other countries. However, Bhatti et al.^13^ found that 1.87% of their transfusion dependant thalassemic patients developed autoantibodies. Another study in Kuwait^10^ reported that 11% of their patients developed autoantibodies. The higher rate found in our study may be explained by non phenotypically blood exposure in some of our patients.^14^

DFO was considered for a long time as the gold standard in iron chelation therapy. It has significantly improved life expectancy and the quality of life of patients with iron overload.^15^ But long-term management of iron overload is suboptimal in many patients, in part because of compliance issues associated with the parenteral administration regimen. Deferasirox, a once-daily oral iron chelator has proven safe and effective in reducing liver iron concentrations and serum ferritin levels in patients with various transfusion-dependant anaemia.^16^,^17^ In our study and surprisingly, treatment with Deferoxamine led to a considerable reduction of mean plasma ferritin levels when administered by subcutaneous bolus injection. However, mean plasma ferritin levels were significantly higher compared to Deferasirox when administered by pump. This might be explained by a lack of chelating treatments in some hospitals in the country and poor compliance among some of the studied patients.

Heart disease may manifest as pulmonary hypertension, arrhythmias, systolic/diastolic dysfunction, pericardial effusion, myocarditis or pericarditis. 19.8% of our studied patients, suffered from heart disease, compared with a prevalence of 15.1% reported among 566 Sicilian thalassemic patients.^18^ Comparison of the prevalence of cardiac involvement with other reports shows that heart failure is higher in Tunisia than in the other countries.. In a cohort of 1146 patients born from 1960 through 1987, Borgna-Pignatti et al^15^ found that the incidence of heart failure by 15 years of age account for 5% of the patients born between 1970 and 1974 and 2% in those born between 1980 and 1984. The reasons are not clear but are probably multiple and include less frequent transfusions, lower pre-transfusion hemoglobin level and inadequate chelation therapy.^19^

Iron-overload associated endocrinopathy is a frequently reported complication in chronically transfused TM patients with 60% of the patients with a dysfunction of at least one gland.^20^,^21^ These include diabetes mellitus (DM), hypogonadism, hypothyroidism, hypoparathyroidism, and low bone mass. Hypogonadotropic hypogonadism remains a common endocrinopathy in multitransfused TM patients.^22^ Our data are consistent with the Italian cohort in which hypogonadism was reported in nearly 50% of cases.^23^ In contrast, the prevalence of growth retardation was higher(26.7%) than that reported from Italy, where it is found in less than 5% of the patients.^19^,^23^ Hypothyroidism, diabetes mellitus and hypoparathyroidism are common particularly for patients in the second decade of life. Our overall diabetes rate of 4.2 % is lower than overall rate reported in Brittenham’s et al.^24^ cohort of 59 patients, aged 7 to 31 years. It is comparable, however, to that reported from Italy, where it is found in less than 5% of the patients^25^. Mean serum ferritin level in TM patients with diabetes and those without diabetes was significantly different. However, no correlation was found between occurrence of diabetes and type chelation therapy.

Splenectomy is also beneficial in treating thalassemia major. It reduces patients’ transfusion requirement and iron overload and increases the main level of haemoglobin.^26^ In our study 54.0% of patients underwent splenectomy. The high number of patients who were already splenectomized at the time of the first survey might indicate that previous transfusion therapy had been inadequate in at least some of them. The risk of invasive bacterial infection in splenectomized patients is well known. Data collected by Bisharat et al.^27^ supports this concept. They reviewed 28 studies amounting to 6942 well-documented patients, 209 of whom developed invasive infection. Subtotal splenectomy may reduce the risk of post-splenectomy sepsis.^28^ Nevertheless, there are not, at the moment, specific recommendations for this procedure which has technical drawbacks in this population including regrowth of the spleen and the need for reoperation.^29^ Streptococcus pneumoniae was responsible for the majority of the infections (66%). It is followed for incidence by H. influenzae type b, Escherichia coli, and Neisseria meningitides. Thus prevention and treatment of bacterial infections in splenectomized thalassemic patients are life-saving measures. Splenectomized patients must receive routine vaccination, including both live attenuated and killed vaccines, but they should also be immunized against Streptococcus pneumoniae, H. influenza type b, and Neisseria meningitides. However, vaccination does not completely protect against infection with encapsulated bacteria and prophylactic antibiotics have a role as well. According to other studies, cardiac failure and rhythm disturbances remain the main causes of death among our patients.^30^,^31^ Severe anemia, if untreated, can result in high-output cardiac failure. Otherwise, cardiac failure may also result from multiple life-long transfusions. In addition infections are a frequent complication of thalassemias and they can be fatal. In our study, infections were the second cause of death after heart failure in polytransfused TM patients. Similar results were reported in Greece and in Italy.^15^,^32^ The analysis of survival rates according to chelation treatment showed that patients treated with oral chelator have a survival rate of 92.2% compared to 66.66% in patients treated with DFO. Interestingly a standard care in UHC was associated with higher rate of survival. This may be explained by several reasons mainly, lack of knowledge, difficulties in follow-up due to low-income of concerned population and unavailability of chelator. To improve the situation, public education about thalassemia is of a great importance and should be carried out through periodic meetings addressed to health professionals including doctors and nurses working in the community, and family members. Also, all means of mass media are helpful as well as the sensitization through patient parents ‘associations that facilitates the contact with families and the diffusion of information through didactic supports (brochures, booklets ect…). In reality the whole problem still lies in the difficulties in the diagnosis of abnormal hemoglobin traits and in the very limited economic resources that do not permit to take in charge correctly the numerous patients already identified. this prevention programme in Sardinia^33^, the incidence of thalassaemia patients has decreased from 1:250 live births to 1:1000 live births. Similarly in Cyprus,^34^ the incidence of thalassaemia major cases dropped by 96%.

## Conclusions

TM must be taken as a public health problem in Tunisia. Series of important conclusions can be drawn. First of all, a centralization of care institutions seems to be necessary. Intensified collaboration between smaller regional hospitals taking care of only a few patients and central medical institutions treating a greater number of patients is desirable. The use of individually adjusted intensification of chelation therapy in connection with suitable strategies for treating siderotic complications must be extended. Finally steps need to be taken to develop preventive measures like premarital screening, genetic counseling and prenatal diagnosis because of the cost of treatment depending on the quality of care.

## Figures and Tables

**Table 1:** Evaluation of mean ferritinemia in treatment groups

Chélation therapy	Ferritinemia (μg/l)	P
DFO	2262	NS
Oral chelator	2150	
DFO	2262	0.006
DFP	2863	
DFX	1622	
DFX	1622	0.000
DFP	2863	
DFX	1622	0.02
DFO	2262	
DFX	1650	0.001
DFO pompe	3074	
DFX	1650	NS
DFO bolus	1635	

DFO	2262	NS
DFP	2863	

DFO: Deferoxamine; DFX: Deferasirox; DFP: Deferiprone; NS: not statistically significant

**Table 2:** Endocrine complications in patients with TM

Endocrinopathy	Total number	Ferritin level			P
		<1000	[1000–2500]	>2500	
Growth retardation					0.000
Yes	54	7	19	28	
No	154	58	65	31	
Delayed puberty					0.009
Yes	33	7	11	15	
No	40	17	16	7	
Hypothyroidism					0.000
Yes	18	0	3	15	
No	244	81	102	61	

Diabetes					0.042
Yes	9	2	1	6	
No	252	77	103	72	

**Table 3:** Mortality related complications

	Alive	Died	p
Cardiopathy			0.000
Yes	39	24	
No	246	9	
Mean ferritin level(μg/l)	1987	3662	0.000
DFO	191	33	0.001
Oral chelator	108	4	
DFO	191	33	0.006
DFX	62	2	
DFO	191	33	0.03
DFP	46	2	
Follow up at UHC			0.003
Yes	296	36	
No	43	15	

Splénectomy			NS
Yes	176	25	
No	155	16	

UHC: university hospital center
